# Surgical management of progressive spinal deformities in FKBP14-associated Ehlers-Danlos syndrome: a case report and literature review

**DOI:** 10.1007/s00381-025-07063-1

**Published:** 2025-12-09

**Authors:** Bluyé DeMessie, Jason Yu, Shanzeh Sayied, Genesis Liriano, Jacob F. Schulz, Andrew J. Kobets

**Affiliations:** 1 Department of Neurological Surgery, Montefiore Medical Center/Albert Einstein College of Medicine, Bronx, NY USA; 2 Department of Orthopedic Surgery, Montefiore Medical Center/Albert Einstein College of Medicine, Bronx, NY USA

**Keywords:** Ehlers-Danlos syndrome, Basilar invagination, Early-onset scoliosis, Pediatric spine

## Abstract

**Purpose:**

This report aims to present a comprehensive case of progressive spinal instability in kyphoscoliotic Ehlers-Danlos syndrome (EDS) and review the surgical management literature to provide insights into treatment strategies for this challenging pediatric population.

**Case presentation:**

A female patient with genetically confirmed FKBP14-associated EDS (homozygous c.362dupG p.E122RfsX7 mutation) was followed from infancy through age 10 at a tertiary pediatric center. At 15 months, rapidly progressive cervical kyphosis with myelopathy necessitated urgent C3 corpectomy with C2–C4 fusion. Progressive early-onset scoliosis required magnetically controlled growing rod placement at age 5. Development of basilar invagination led to occipital-cervical fusion at age 7, followed by growing rod exchange at age 8. Modified fixation techniques accommodating tissue fragility were employed throughout. Nine-year follow-up demonstrated successful deformity control with solid arthrodesis at multiple levels, though ongoing challenges include hardware adaptation and the need for mobility assistance.

**Conclusion:**

This case demonstrates that while EDS presents progressive challenges requiring reactive surgical interventions, early recognition and timely response to evolving deformities can achieve favorable long-term outcomes. Our experience highlights the importance of regular surveillance to detect new pathology before irreversible complications occur.

## Introduction

The Ehlers-Danlos syndromes (EDS) comprise a genetically diverse family of inherited connective tissue disorders characterized by a constellation of clinical manifestations including joint hypermobility, excessive skin elasticity, recurrent joint subluxations, ligamentous laxity, persistent pain syndromes, urogenital dysfunction, and fragility of tissues and blood vessels [1, 2]. Signs and symptoms of EDS may manifest during infancy, yet definitive diagnosis typically occurs in adolescence or early adulthood due to the complex nature of phenotypic expression and overlapping features with other connective tissue disorders [2]. Spinal manifestations of EDS include craniocervical instability (CCI), Chiari malformation I, spinal deformity, CSF leaks, tethered cord syndrome, and musculoskeletal problems [1, 3–5]. In pediatric EDS, these deformities may be asymptomatic until weight-bearing stresses increase on the connective tissues as the child grows. Thus, early conservative management or surgical intervention is critical to prevent the progression of symptomatology and spinal deformities that interfere with patient quality of life.

The 19 known pathogenic genetic variants causing EDS manifest either as abnormalities in collagen or as deficits in post-translational collagen processing [1, 6]. Kyphoscoliotic EDS (type VI) represents an uncommon variant characterized by profound congenital muscle hypotonia, early-onset progressive kyphoscoliosis, joint hypermobility, and sensorineural hearing impairment present from birth [7, 8]. Early diagnosis and surgical intervention are often required to treat kyphoscoliotic EDS since progression of the curvature occurs quickly and conservative management strategies such as bracing are often ineffective [1]. Kyphoscoliotic EDS demonstrates autosomal recessive inheritance and results from either deficiency of lysyl hydroxylase 1 due to mutations in PLOD1 or alterations in FKBP14, which encodes a peptidyl-prolyl cis–trans isomerase that functions as a molecular chaperone for multiple collagen types (III, IV, VI, and X) [9]. The patient described in this report exhibits a homozygous mutation in the FKBP14 gene.

## Case report

**Fig. 1:**
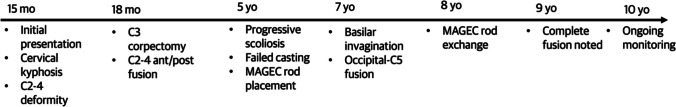
Disease progression timeline

**Fig. 2:**
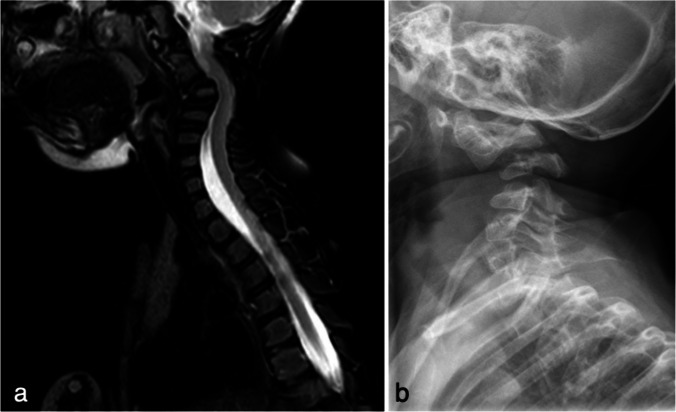
** a** Sagittal T2-weighted MRI at age 15 months reveals cervical kyphosis with ventral compression of the spinal cord by the hypoplastic C4 vertebral body. **b** Lateral cervical radiograph reveals the bony anomalies, underdeveloped vertebral, and indication of ligamentous laxity. The patient underwent C3 corpectomy with C2–C4 anterior and posterior fusion and was previously reported [10]

A female infant with severe EDS type VI, specifically a biallelic mutation of the FKBP14 gene (c.362dupG p.E122RfsX7), initially presented at 15 months of age with regression in gross motor skills (Fig. 1). Initial imaging revealed cervical kyphosis with a 45° deformity at C2–C4 and spinal cord compression (Fig. 2a, b). The patient underwent C3 corpectomy with C2–C4 anterior and posterior fusion using specialized craniofacial miniplates. Given the acute neurological symptoms with motor regression and the severity of compression seen on imaging, halo gravity traction was considered but deemed inappropriate due to the urgent need for decompression. The rapid progression of symptoms necessitated immediate surgical intervention. This initial procedure was previously reported [10] and achieved solid fusion with maintenance of correction.

**Fig. 3:**
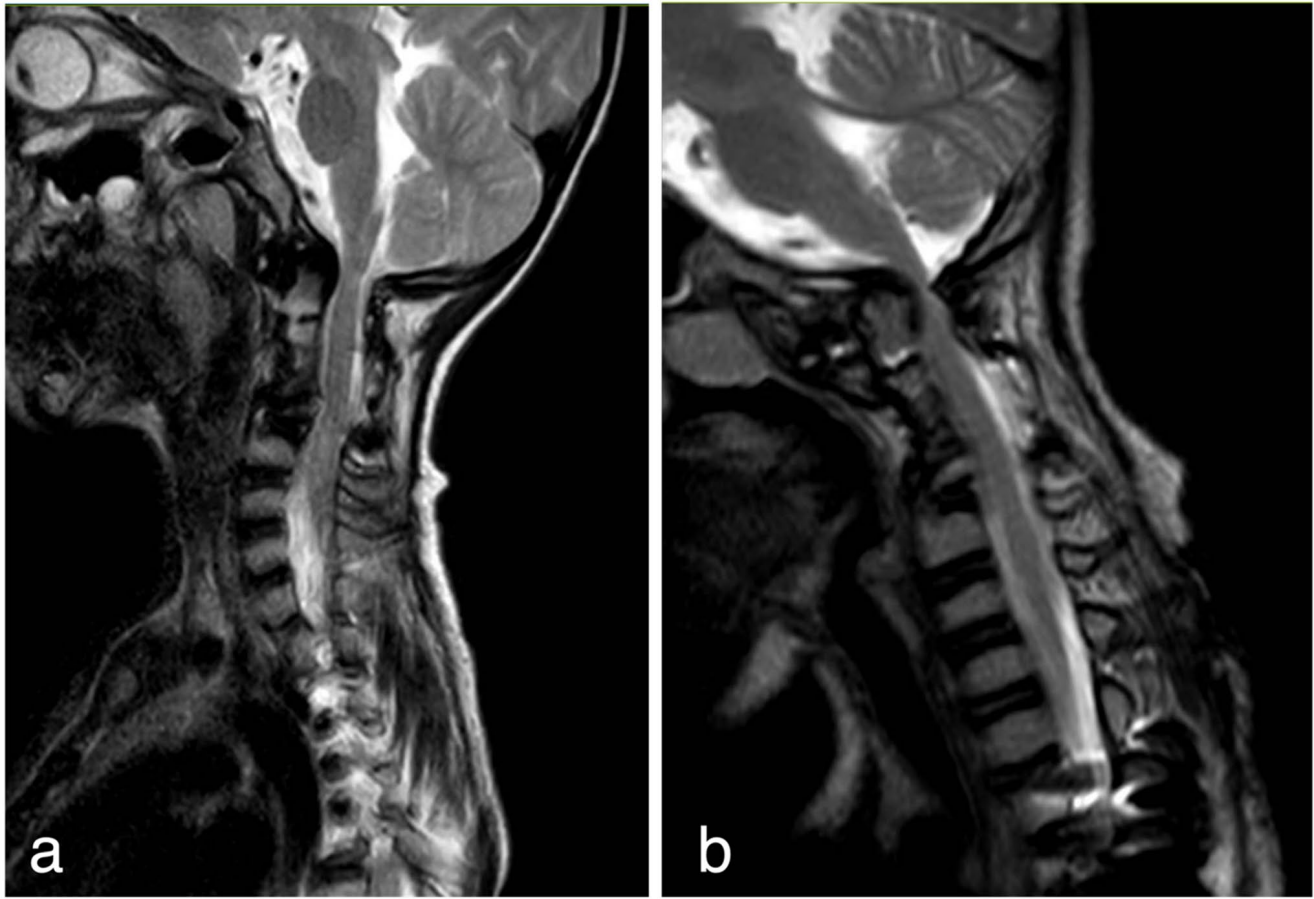
**a** Sagittal T2-weighted MRI at age 5 demonstrating initial mild odontoid protrusion through foramen magnum in a patient with Ehlers-Danlos syndrome. **b** Follow-up sagittal T2-weighted MRI at age 7 showing progression to severe basilar invagination with brainstem impingement, necessitating urgent surgical decompression and fusion

At age 5, baseline imaging demonstrated increased dorsal angulation of the dens with mild protrusion into the foramen magnum and interval narrowing of the spinal canal at C1–C2 (Fig. 3a). The pre-dens space measured approximately 3–4 mm. Moderate dextroscoliosis of the thoracic spine and levoscoliosis of the lumbar spine were noted, along with congenital spinal canal stenosis throughout the spine. Following failed conservative management with casting for progressive early-onset scoliosis, the patient underwent posterior spinal fusion of T2–T4 and L3–L4 with placement of magnetically controlled growing rods. Following general anesthesia with endotracheal intubation, baseline motor and sensory evoked potentials were established. The patient was positioned prone on a flat Jackson table with special attention to neck positioning given her previous cervical fusion.

The surgical approach involved two separate incisions: T2–T4 and L3–L4. Following exposure, pedicle screws were placed at the anchor sites using C-arm navigation and freehand technique. Screws of 4.5 mm diameter were used proximally and 5-mm screws distally. The surgical team utilized a MAGnetic Expansion Control (MAGEC) system with 5-mm-diameter rods and 90-mm actuators to enable controlled spinal growth. A submuscular tunnel was created using chest tubes as guides for rod passage. The rods were secured to the pedicle screws, and gentle distraction was performed on the concave side with continuous neuromonitoring. Local autograft and 30 mL of cancellous chips were placed at the fusion sites. The MAGEC system employs an external remote controller that generates a rotating magnetic field, causing the internal magnet within the rod to rotate and extend the telescopic mechanism. In the outpatient setting, lengthening is performed non-invasively by placing the external controller over the implanted rod for approximately 2–3 min, typically achieving 3–5 mm of distraction per session.

At age 7, surveillance imaging revealed progression of basilar invagination with significant spinal canal and foramen magnum stenosis (Fig. 3b), though the patient remained clinically stable without acute neurological symptoms. The thoracolumbar spine showed moderate S-shaped scoliosis with fusion from T2 to T4 and L3 to L4, maintained by growing rods. The C2–C4 fusion demonstrated osseous integration, though subsequent imaging revealed C3 facet screw loosening identified on surveillance imaging. Neuroimaging showed high-grade bilateral stenosis of the neuroforamina at C2–C3 and C3–C4 levels, while the lower cervical segments remained relatively preserved.

**Fig. 4:**
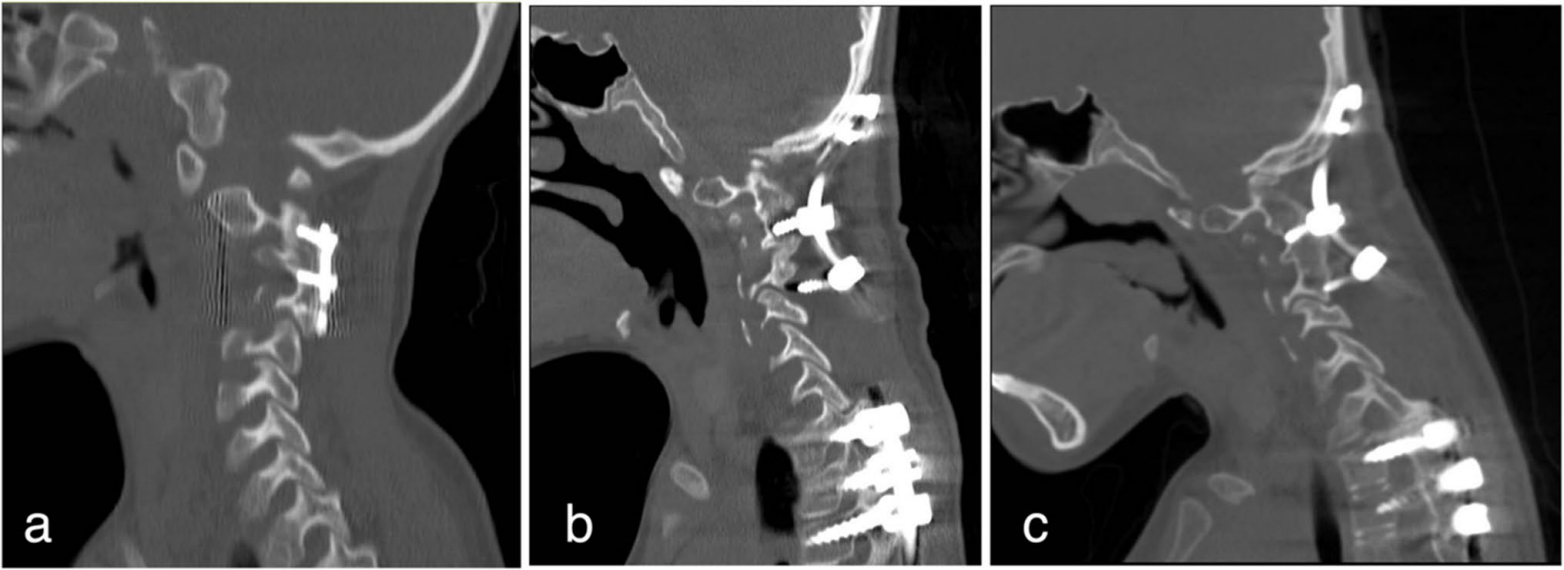
**a** Cervical spine CT at age 3 showing initial C2–C4 anterior–posterior fusion with anterior plating and posterior instrumentation, moderate kyphosis, and complete facet fusion. **b** Cervical spine CT at age 8 demonstrating occipital-cervical fusion with occipital plating and instrumentation to C4, screw loosening, and basilar invagination. **c** Cervical spine CT at age 9 revealing complete C2–C4 vertebral body fusion and C1–C5 posterior fusion with resected C2–C5 spinous processes

Given the significant radiographic findings despite minimal symptoms, the patient was managed prophylactically with a hard cervical collar while surgical intervention was planned. Halo gravity traction was considered but not pursued due to concerns about the severe connective tissue fragility in EDS potentially leading to pin site complications and the established basilar invagination pattern that was unlikely to significantly reduce with traction alone. This case highlights the importance of balancing radiographic progression against clinical status in the management of CCI, particularly in patients with connective tissue disorders. Initial imaging demonstrated moderate kyphosis with anterior plating and posterior instrumentation, with complete facet fusion at C2–C3 and C3–C4 levels and patent interbody spaces (Fig. 4a).

After thorough preoperative optimization, the patient underwent occipital-to-C5 fusion with suboccipital decompression and C1 laminectomy. The procedure involved a NuVasive occipital keel plate secured with three 4.5-mm-diameter screws and five pedicle screws in the cervical spine, connected by two 3.5-mm titanium rods (Fig. 4b). Sequential compression and distraction achieved indirect reduction of foramen magnum stenosis. The construct was augmented with autologous rib graft extending from the occipital plate to the C2–C4 fusion mass. This approach achieved adequate decompression of the brainstem and stabilization of the craniocervical junction (CCJ) while preserving neurological function.

At age 8, mechanical failure of the internal lengthening mechanism was identified during routine outpatient lengthening attempts, with inability to achieve further distraction despite appropriate external magnet application, necessitating rod exchange. Through the previous incisions, the T2–T4 and L3–L4 regions were exposed. The solid arthrodesis at the anchor points was confirmed. Using a 28-French chest tube as a guide, each rod was carefully removed and replaced with new MAGEC rods. The rods were secured proximally and distally with set screws, with radiographic confirmation of position and deformity correction.

Follow-up imaging through ages 8–9 demonstrated evolution of the fusion constructs. Recent studies showed complete bony fusion of the C2–C4 vertebral bodies and C1–C5 posterior elements (Fig. 4c). Despite initial appropriate positioning of all screws, underlying compromised bone mineralization and structural integrity led to some hardware loosening, particularly around the C3–C5 facet screws. However, the combination of halo-vest immobilization and biological healing promoted adequate fusion, precluding the need for hardware revision.

Radiological surveillance revealed gradual stabilization of the fusion constructs, though with some hardware-related complications. Sequential imaging showed progressive bony fusion at the surgical sites, with complete integration noted at the C2–C4 vertebral bodies and C1–C5 posterior elements by age 9, despite persistent C3–C5 facet screw loosening without clinical consequence due to solid bony fusion.

Regular outpatient evaluations revealed stable neurological status with some persistent functional limitations. The patient maintained baseline strength of 4/5 in upper extremities and varying degrees of strength in lower extremities, ranging from 3/5 in knee extension to 4/5 in ankle movements. She developed a scissoring gait pattern and requires assistance for ambulation, typically using a wheelchair for mobility.

**Fig. 5:**
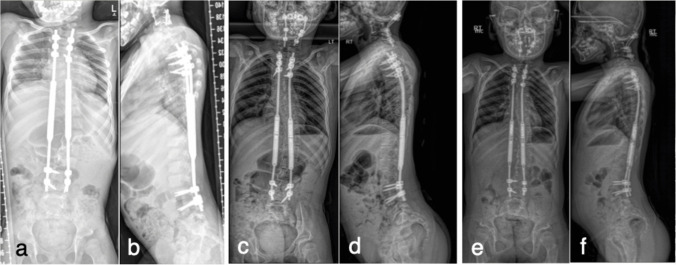
**a** Full spine radiographs at age 5 years 7 months showing moderate S-shaped thoracolumbar scoliosis with initial MAGEC rod placement from T2–T4 to L3–L4, status post C2–C4 fusion. **b** Age 8 years 10 months demonstrating maintained alignment with exchanged growing rods, progression of basilar invagination, and new occipital-to-C5 fusion construct. **c** Age 9 years 10 months revealing stable constructs with appropriate interval lengthening and no hardware complications

Genetic evaluation confirmed the diagnosis of EDS, kyphoscoliotic and deafness type, with the homozygous pathogenic variant reflecting parental consanguinity. The patient exhibits characteristic features including significant ligamentous laxity in all joints and bilateral knee valgus/varus instability. The MAGEC rod system has required ongoing management with regular lengthening procedures performed in the outpatient setting. These procedures have generally been well tolerated, though some mechanical challenges have been noted, including mild rod kickback. Serial imaging has demonstrated maintained correction without evidence of hardware failure or adjacent segment degeneration (Fig. 5a–c). The patient has experienced intermittent paraspinal neck pain, managed conservatively with massage and topical treatments. This symptomatology appears to be multifactorial and may relate to hardware prominence, though removal of cervical implants has not been necessary. She participates in regular physical and occupational therapy, supplemented by aquatic exercises. Functionally, the patient has adapted well, pursuing home-based education with plans for transition to traditional schooling. She maintains regular follow-up with multiple subspecialties, including orthopedics, physical medicine and rehabilitation, and genetics. Her care team has implemented comprehensive management strategies, including bracing (AFOs), adaptive equipment, and regular therapeutic interventions.

## Scoping review of pediatric fkbp14-related EDS with spinal involvement

To contextualize our patient’s presentation within the broader spectrum of FKBP14-related EDS, we performed a literature review identifying 31 pediatric cases with documented spinal involvement across 5 major case series [8, 9, 11–13] (Table 1). Among these cases, 71% (22/31) developed spinal deformity within the first year of life, with kyphoscoliosis being the predominant pattern. Surgical intervention was reported in 35% (11/31) of cases, with procedures ranging from single-stage posterior fusion to complex craniocervical stabilization. Notably, CCI requiring surgical intervention was documented in only two cases (6%), both presenting after age 4 years. Our patient’s clinical course, requiring four major spinal procedures between ages 15 months and 8 years, represents one of the most extensive surgical interventions reported in the literature for FKBP14-related EDS.

**Table 1:** Published cases of pediatric FKBP14-related Ehlers-Danlos syndrome with spinal involvement

Study/year	Patient ID	Sex/age at report	Mutation(s)	Age at spinal onset	Spinal deformity type	Surgical intervention	Other features	Outcome/follow-up
Baumann et al. 2012 [11]	P1	M/16y	c.362dupC (hom)	2 mo	Progressive kyphoscoliosis	Dorsal thoracolumbar spondylodesis (age 8y)	Severe hypotonia from birth, hearing loss	FVC improved 47% to 66%, 9 cm height gain
	P2	F/1y	c.362dupC (hom)	< 1y	Kyphoscoliosis	Not reported	Severe hypotonia, delayed motor	Early presentation
	P3	F/12y	c.558delT (hom)	< 1y	Progressive kyphoscoliosis	Not reported	Joint hypermobility, myopathy	Stable at report
	P4	M/13y	c.362dupC (hom)	Infancy	Kyphoscoliosis	Not reported	Hearing loss, hypotonia	Progressive course
	P5	F/7y	c.299_300insA (hom)	Early infancy	Progressive kyphoscoliosis	Not reported	Muscle weakness, joint hypermobility	Progressive deformity
	P6	M/9y	c.375G > A/c.620G > T	< 1y	Severe kyphoscoliosis	Surgical stabilization planned	Congenital hearing impairment, myopathy	Wheelchair dependent
Giunta et al. 2018 [12]	FI	M/17y	c.362dupC (hom)	Birth	Severe progressive kyphoscoliosis	Multiple surgeries	Congenital muscle hypotonia, hearing loss	Wheelchair bound
	FII	F/9y	c.362dupC (hom)	Early infancy	Severe progressive scoliosis	Surgical correction (age not specified)	Walked at 7y, severe hypotonia	Postoperative improvement
	FIII	M/4y	c.362dupC (hom)	4y	Atlantoaxial subluxation, myelocompression	Posterior occipitocervical fixation	Dens dislocation, hearing loss	Stable at 9y follow-up
	FIV-1	F/4y	c.197 + 5_197 + 8del (hom)	Infancy	Kyphoscoliosis	Not reported	Premature (31w), respiratory support	Ongoing management
	FIV-2	M/6y	c.197 + 5_197 + 8del (hom)	< 2.5y	Severe kyphoscoliosis	Surgical correction at 2.5y	Less severe than sibling	Improved post-surgery
	FV	F/3y	c.362dupC (hom)	Infancy	Mild scoliosis	Not required	Hypotonia, joint laxity	Stable, conservative management
	FVI	M/8y	c.362dupC (hom)	< 1y	Progressive kyphoscoliosis	Under consideration	Hearing loss, myopathy	Progressive deformity
	FVII	F/14y	c.587A > G (hom)	6 mo	Severe kyphoscoliosis	Planned	Never walked independently	Wheelchair dependent
	FVIII	M/12y	c.362dupC (hom)	< 1y	Progressive scoliosis	Not reported	Developmental delay	Ambulatory
	FIX	F/10y	c.431_442del (hom)	Early childhood	Kyphoscoliosis	Conservative	Joint hypermobility	Stable
	FX	M/15y	c.362dupC (hom)	Infancy	Severe scoliosis	Posterior fusion at 13y	Hearing loss	Good surgical outcome
	FXV	F/11mo	c.439C > T/c.497C > T	9 mo	Mild thoracolumbar scoliosis	Not required	Earliest radiographic documentation	Early detection
	FIII-2	F/2y	c.362dupC (hom)	Infancy	Progressive kyphoscoliosis	Not reported	Hypotonia, delayed motor milestones	Under observation
	FXI	M/11y	c.320A > G (hom)	Early childhood	Moderate kyphoscoliosis	Conservative management	Joint hypermobility, myopathy	Stable
	FXII	F/8y	c.362dupC (hom)	< 1y	Progressive kyphoscoliosis	Bracing	Muscle weakness, hearing impairment	Progressive
	FXIII	M/5y	c.614 T > C (hom)	2y	Mild scoliosis	Not required	Hypermobile joints, delayed walking	Ambulatory
	FXIV	F/13y	c.362dupC (hom)	Infancy	Severe kyphoscoliosis	Spinal fusion at 11y	Never walked, severe hypotonia	Wheelchair dependent
	FXVI	M/10y	c.299_300insA (hom)	Early infancy	Progressive kyphoscoliosis	Under consideration	Congenital hearing loss, myopathy	Progressive deformity
Dordoni et al. 2016 [8]	Case 1	M/8y	c.362dupC/c.573_575del	Early childhood	Mild non-progressive kyphoscoliosis	Not required	Arterial rupture at 6y, atlantoaxial instability	Vascular monitoring
Castori et al. 2019 [13]	Case 1	F/15y	c.362dupC (hom)	Infancy	Severe kyphoscoliosis, occipitoatlantoaxial instability	Not specified	Never walked, hip dysplasia, osteopenia	Severe phenotype
Colman et al. 2022 [9]	Proband 1	F/7y	c.587A > G (hom)	Early childhood	Progressive kyphoscoliosis	Conservative	Walked at 2y, joint laxity, microcornea	Ambulatory
	Proband 2	F/8y	c.362dupC (hom)	3 mo	Severe progressive kyphoscoliosis	Not reported	Severe hypotonia, strabismus	Progressive
	Proband 3	F/5y	c.2 T > G (hom)	Infancy	Progressive kyphoscoliosis	Under consideration	Pronounced joint flexibility, microcornea	Ongoing management

*EDS* Ehlers-Danlos syndrome, *EOS* early-onset scoliosis, *F* female, *FVC* forced vital capacity, *hom* homozygous, *M* male, *mo* months, *y* years

## Discussion

Surgical management of pediatric patients with EDS presents unique challenges due to connective tissue abnormalities [14]. These patients demonstrate increased tissue fragility, making both skin and internal organs vulnerable to shear forces during procedures. Careful positioning and adequate padding are essential to minimize intraoperative tissue damage. EDS patients also experience delayed wound healing, higher infection rates, and increased bleeding risk [15]. Postoperative care requires meticulous wound monitoring, use of non-adhesive dressings, and consideration of prophylactic antibiotics [3, 16].

CCI management in EDS patients often requires occipital-cervical fixation (OCF). While traditional approaches utilize occipital plate fixation, the thin occipital bone often seen in pediatric EDS patients may provide inadequate purchase for standard plating systems. Therefore, EDS patients may necessitate occipital condyle screw fixation. This alternative technique, while providing strong anchoring, significantly reduces rotational range and presents technical challenges, particularly in pediatric patients with diminutive anatomy [3]. Post-OCF fusion, EDS patients demonstrate higher rates of hardware failure compared to non-EDS populations due to inadequate ligamentous and soft tissue support, with an increased likelihood of requiring revision surgery within 2 years [17].

Timing surgical interventions in young EDS patients requires careful consideration of growth potential. While early intervention can address structural deformities, premature surgery may interfere with normal bone development by restricting growth plate movement [18]. However, severe conditions such as congenital kyphoscoliosis in EDS type VI, presenting before age one, may require immediate intervention despite growth concerns to prevent progression to restrictive lung disease and neurological complications [9].

Comprehensive multidisciplinary treatment is essential for managing EDS, with conservative approaches preferred due to surgical risks from tissue fragility [19, 20]. Management strategies focus on joint instability, muscle weakness, and mobility issues. Bracing provides support for weakened ligaments and tendons, while casting is limited to acute settings due to tissue risks [20]. Physical therapy aims to improve muscle strength, proprioception, and joint stability through isometric exercises and closed kinetic chain movements [21, 22]. Therapy programs are individualized given the variable presentation of EDS [15].

For asymptomatic patients, evidence supports clinical surveillance over prophylactic intervention, with biannual examinations including Beighton scoring and neurological assessment [19, 23]. Radiographic studies are obtained at diagnosis and repeated based on clinical indications rather than at fixed intervals [24]. While some clinicians advocate early surgical management to prevent progression, others maintain that conservative treatment with physical therapy and monitoring is appropriate until clear clinical or radiographic deterioration occurs [19, 20]. Current guidelines suggest considering surgical intervention when thoracic curves exceed 50° or demonstrate annual progression greater than 10°, though evidence supporting these thresholds remains limited [24]. The paucity of comparative studies between early intervention and conservative management highlights a critical knowledge gap in optimal treatment timing [19].

Our scoping review (Table 1) reveals considerable heterogeneity in the spinal manifestations and management approaches for FKBP14-related EDS. While our patient required extensive surgical intervention beginning at 15 months, the majority of reported cases (65%) were managed conservatively or with less extensive procedures. This variability likely reflects differences in disease severity, institutional practices, and thresholds for surgical intervention. The rarity of reported CCI (6% in our review) makes our patient’s progression from cervical kyphosis to basilar invagination particularly notable, though this may represent under-recognition rather than true rarity. These findings underscore that treatment decisions must be individualized based on each patient’s specific manifestations and progression patterns rather than following a standardized protocol.

Surgical intervention is considered when conservative management fails or severe dysfunction develops [19]. Indications include significant joint instability requiring arthrodesis or realignment [25] and progressive spinal curves causing functional impairment [16]. In young patients, surgical timing must balance growth preservation against preventing irreversible damage [26, 27]. The decision for early versus delayed intervention depends on EDS subtype, functional status, and quality-of-life considerations [7].

CCI in EDS results from ligamentous laxity at the CCJ, potentially causing neurological symptoms through neural compression [3]. While not all EDS patients develop neurological injury from CCI, severe cases may require surgical intervention to prevent permanent deficits [5, 28]. While morphometric measurements such as the basion-axial interval have been described, their reliability in young children with connective tissue disorders remains uncertain [5]. In our case, clinical progression and direct visualization of brainstem compression on MRI guided intervention timing. Initial management typically involves conservative measures like physical therapy and cervical orthosis. For severe cases, internal OCF is performed to stabilize the CCJ and correct misalignments [29]. Rigid fixation with screws and plates is preferred over semi-rigid methods for better biomechanical stability [30].

Pediatric cases present unique challenges due to abnormal anatomy and small vertebral dimensions [31]. In young children with inadequate bone stock for rigid fixation, alternative techniques including wiring and rib allografts have been described, though modern image guidance increasingly allows for screw placement even in challenging pediatric cases [32, 33]. For spinal deformity management, growth-friendly instrumentation options include traditional growing rods and magnetically controlled growing rods [34].

A coordinated multi-disciplinary approach was essential for managing this complex case of severe EDS with early-onset scoliosis and CCI. The core surgical team integrated pediatric orthopedic surgery, neurosurgery, and plastic surgery expertise for complex spinal procedures. Plastic surgery provided critical support in soft tissue management and autologous grafting. The perioperative team included specialized pediatric anesthesiologists, neurophysiological monitoring specialists, and intensive care physicians. Genetic confirmation of FKBP14-related EDS established the diagnosis and indicated a high risk for progressive deformity, supporting close surveillance protocols rather than directly altering surgical technique. Rehabilitation services were coordinated through physical medicine specialists, with twice-weekly physical and occupational therapy sessions at a pediatric rehabilitation center, supplemented by aquatic therapy. Monitoring protocols included initial CT and MRI studies for CCJ assessment, with subsequent EOS full spine radiographs for routine follow-up. The MAGEC system required outpatient lengthening every 3 months, with radiographic confirmation of proper rod distraction and alignment. Rehabilitation strategies adapted to post-surgical needs while maintaining mobility and preventing contractures. Adaptive equipment, including a power-assist wheelchair and ankle–foot orthoses, supported functional independence. This framework demonstrates the value of coordinated multi-disciplinary care in managing complex EDS cases, offering a potential model for similar cases.

Based on our experience, we recommend the following surveillance protocol for pediatric EDS patients with spinal involvement: clinical evaluation every 3 months with focused neurological examination, EOS full spine radiographs every 6 months during periods of rapid growth (ages 0–5 and 10–15 years) and annually during slower growth periods, and MRI annually or with any clinical changes suggesting neural compression. More frequent imaging may be warranted following surgical interventions or with symptom progression. Additional research is needed to establish evidence-based guidelines for surgical timing and to better understand the long-term impact of interventions on patient outcomes.

## Conclusion

This case of severe Ehlers-Danlos syndrome, kyphoscoliotic and deafness type, demonstrates successful management of complex pediatric spinal deformity through coordinated multidisciplinary care. Key interventions included strategic timing of surgical procedures, particularly the implementation of magnetically controlled growing rods and occipital-cervical fusion. Success factors included modified fixation techniques appropriate for connective tissue disorders, comprehensive neurophysiological monitoring, and regular imaging surveillance that enabled timely adjustments to treatment. The case highlights several areas requiring further investigation: optimization of the MAGEC rod system to reduce mechanical complications, exploration of alternative growth-friendly implants, development of biological treatments targeting underlying genetic causes, and assessment of long-term functional outcomes in EDS patients. While managing complex EDS cases presents significant challenges, this case illustrates that favorable outcomes are achievable through individualized surgical approaches and sustained multidisciplinary care.

## Data Availability

No datasets were generated or analysed during the current study.

## References

[1] Marathe N, Lohkamp LN, Fehlings MG (2022) Spinal manifestations of Ehlers-Danlos syndrome: a scoping review. J Neurosurg Spine 37:783–793 35986728 10.3171/2022.6.SPINE211011

[2] Feldman ECH, Hivick DP, Slepian PM, Tran ST, Chopra P, Greenley RN (2020) Pain symptomatology and management in pediatric Ehlers-Danlos syndrome: a review. Children. 10.3390/children7090146 32967103 10.3390/children7090146 PMC7552757

[3] Mao G, Kopparapu S, Jin Y, Davidar AD, Hersh AM, Weber-Levine C, Theodore N (2022) Craniocervical instability in patients with Ehlers-Danlos syndrome: controversies in diagnosis and management. Spine J 22:1944–1952 36028216 10.1016/j.spinee.2022.08.008

[4] Stern CM, Pepin MJ, Stoler JM, Kramer DE, Spencer SA, Stein CJ (2017) Musculoskeletal conditions in a pediatric population with Ehlers-Danlos syndrome. J Pediatr 181:261–266 27908650 10.1016/j.jpeds.2016.10.078

[5] Henderson FC Sr, Austin C, Benzel E, Bolognese P, Ellenbogen R, Francomano CA, Ireton C, Klinge P, Koby M, Long D, Patel S, Singman EL, Voermans NC (2017) Neurological and spinal manifestations of the Ehlers-Danlos syndromes. Am J Med Genet C Semin Med Genet 175:195–211 28220607 10.1002/ajmg.c.31549

[6] Malfait F, Francomano C, Byers P, Belmont J, Berglund B, Black J, Bloom L, Bowen JM, Brady AF, Burrows NP, Castori M, Cohen H, Colombi M, Demirdas S, De Backer J, De Paepe A, Fournel-Gigleux S, Frank M, Ghali N, Giunta C, Grahame R, Hakim A, Jeunemaitre X, Johnson D, Juul-Kristensen B, Kapferer-Seebacher I, Kazkaz H, Kosho T, Lavallee ME, Levy H, Mendoza-Londono R, Pepin M, Pope FM, Reinstein E, Robert L, Rohrbach M, Sanders L, Sobey GJ, Van Damme T, Vandersteen A, van Mourik C, Voermans N, Wheeldon N, Zschocke J, Tinkle B (2017) The 2017 international classification of the Ehlers-Danlos syndromes. Am J Med Genet C Semin Med Genet 175:8–26 28306229 10.1002/ajmg.c.31552

[7] Giunta C, Rohrbach M, Fauth C, Baumann M (1993) FKBP14 Kyphoscoliotic Ehlers-Danlos syndrome. In: Adam MP, Feldman J, Mirzaa GM, Pagon RA, Wallace SE, Amemiya A (eds) GeneReviews. University of Washington, Seattle, Seattle (WA). https://pubmed.ncbi.nlm.nih.gov/31132235/31132235

[8] Dordoni C, Ciaccio C, Venturini M, Calzavara-Pinton P, Ritelli M, Colombi M (2016) Further delineation of FKBP14-related Ehlers-Danlos syndrome: a patient with early vascular complications and non-progressive kyphoscoliosis, and literature review. Am J Med Genet A 170:2031–2038 27149304 10.1002/ajmg.a.37728

[9] Colman M, Vroman R, Dhooge T, Malfait Z, Symoens S, Burnyté B, Nampoothiri S, Kariminejad A, Malfait F, Syx D (2022) Kyphoscoliotic Ehlers-Danlos syndrome caused by pathogenic variants in FKBP14: further insights into the phenotypic spectrum and pathogenic mechanisms. Hum Mutat 43:1994–2009 36054293 10.1002/humu.24456

[10] Kobets AJ, Komlos D, Houten JK (2018) Congenital cervical kyphosis in an infant with Ehlers-Danlos syndrome. Childs Nerv Syst 34:1411–1415 29450629 10.1007/s00381-018-3750-9

[11] Baumann M, Giunta C, Krabichler B, Rüschendorf F, Zoppi N, Colombi M, Bittner RE, Quijano-Roy S, Muntoni F, Cirak S, Schreiber G, Zou Y, Hu Y, Romero NB, Carlier RY, Amberger A, Deutschmann A, Straub V, Rohrbach M, Steinmann B, Rostásy K, Karall D, Bönnemann CG, Zschocke J, Fauth C (2012) Mutations in FKBP14 cause a variant of Ehlers-Danlos syndrome with progressive kyphoscoliosis, myopathy, and hearing loss. Am J Hum Genet 90:201–216 22265013 10.1016/j.ajhg.2011.12.004 PMC3276673

[12] Giunta C, Baumann M, Fauth C, Lindert U, Abdalla EM, Brady AF, Collins J, Dastgir J, Donkervoort S, Ghali N, Johnson DS, Kariminejad A, Koch J, Kraenzlin M, Lahiri N, Lozic B, Manzur AY, Morton JEV, Pilch J, Pollitt RC, Schreiber G, Shannon NL, Sobey G, Vandersteen A, van Dijk FS, Witsch-Baumgartner M, Zschocke J, Pope FM, Bönnemann CG, Rohrbach M (2018) A cohort of 17 patients with kyphoscoliotic Ehlers-Danlos syndrome caused by biallelic mutations in FKBP14: expansion of the clinical and mutational spectrum and description of the natural history. Genet Med 20:42–54 28617417 10.1038/gim.2017.70 PMC5763155

[13] Castori M, Fiorillo C, Agolini E, Sacco M, Minetti C, Novelli A, Guglielmi G, Bertini E (2019) Primary muscle involvement in a 15-year-old girl with the recurrent homozygous c.362dupC variant in FKBP14. Am J Med Genet A 179:317–321 30561154 10.1002/ajmg.a.61006

[14] Wiesmann T, Castori M, Malfait F, Wulf H (2014) Recommendations for anesthesia and perioperative management in patients with Ehlers-Danlos syndrome(s). Orphanet J Rare Dis 9:109 25053156 10.1186/s13023-014-0109-5 PMC4223622

[15] Miklovic T, Sieg VC (2024) Ehlers-Danlos Syndrome. StatPearls Publishing, Treasure Island (FL), StatPearls

[16] Joseph AW, Joseph SS, Francomano CA, Kontis TC (2018) Characteristics, diagnosis, and management of Ehlers-Danlos syndromes: a review. JAMA Facial Plast Surg 20:70–75 29121166 10.1001/jamafacial.2017.0793

[17] Chi J, Raso J, Tadepalli V, Labaran L, Oh E, Wang J, Shen FH, Li X (2024) Outcomes following anterior cervical discectomy and fusion in patients with Ehlers-Danlos syndrome. Glob Spine J 14:1699–1705 10.1177/21925682231151924 PMC11268287 36645101

[18] Heim P, Raghunath M, Meiss L, Heise U, Myllylä R, Kohlschütter A, Steinmann B (1998) Ehlers-Danlos syndrome type VI (EDS VI): problems of diagnosis and management. Acta Paediatr 87:708–710 9686670 10.1080/080352598750014184

[19] Malfait F, Castori M, Francomano CA, Giunta C, Kosho T, Byers PH (2020) The Ehlers-Danlos syndromes. Nat Rev Dis Primers 6:64 32732924 10.1038/s41572-020-0194-9

[20] Song B, Yeh P, Nguyen D, Ikpeama U, Epstein M, Harrell J (2020) Ehlers-Danlos syndrome: an analysis of the current treatment options. Pain Phys 23:429–438 32709178

[21] Garreth Brittain M, Flanagan S, Foreman L, Teran-Wodzinski P (2024) Physical therapy interventions in generalized hypermobility spectrum disorder and hypermobile Ehlers-Danlos syndrome: a scoping review. Disabil Rehabil 46:1936–1953 37231592 10.1080/09638288.2023.2216028

[22] Reychler G, De Backer MM, Piraux E, Poncin W, Caty G (2021) Physical therapy treatment of hypermobile Ehlers-Danlos syndrome: a systematic review. Am J Med Genet A 185:2986–2994 34145717 10.1002/ajmg.a.62393

[23] Simmonds JV (2021) Advances in assessment of hypermobility-related disorders. Am J Med Genet C Semin Med Genet 187:453–457 34741798 10.1002/ajmg.c.31943

[24] Ng SY, Bettany-Saltikov J (2017) Imaging in the diagnosis and monitoring of children with idiopathic scoliosis. Open Orthop J 11:1500–1520 29399226 10.2174/1874325001711011500 PMC5759132

[25] Homere A, Bolia IK, Juhan T, Weber AE, Hatch GF (2020) Surgical management of shoulder and knee instability in patients with Ehlers-Danlos syndrome: joint hypermobility syndrome. Clin Orthop Surg 12:279–285 32904109 10.4055/cios20103 PMC7449847

[26] Mackel CE, Jada A, Samdani AF, Stephen JH, Bennett JT, Baaj AA, Hwang SW (2018) A comprehensive review of the diagnosis and management of congenital scoliosis. Childs Nerv Syst 34:2155–2171 30078055 10.1007/s00381-018-3915-6

[27] Johnson AN, Lark RK (2024) Current concepts in the treatment of early onset scoliosis. J Clin Med. 10.3390/jcm13154472 39124741 10.3390/jcm13154472 PMC11313220

[28] Brodbelt AR, Flint G (2017) Ehlers danlos, complex Chiari and cranio-cervical fixation: how best should we treat patients with hypermobility? Br J Neurosurg 31:397–398 28961036 10.1080/02688697.2017.1386282

[29] Wolfla CE (2006) Anatomical, biomechanical, and practical considerations in posterior occipitocervical instrumentation. Spine J 6:225s–232s 17097542 10.1016/j.spinee.2006.09.001

[30] Martinez-Del-Campo E, Turner JD, Kalb S, Rangel-Castilla L, Perez-Orribo L, Soriano-Baron H, Theodore N (2016) Occipitocervical fixation: a single surgeon’s experience with 120 patients. Neurosurgery 79:549–560 27428783 10.1227/NEU.0000000000001340

[31] Menezes AH (2008) Specific entities affecting the craniocervical region: Down’s syndrome. Childs Nerv Syst 24:1165–1168 18421463 10.1007/s00381-008-0603-y

[32] Joaquim AF, Osorio JA, Riew KD (2020) Occipitocervical fixation: general considerations and surgical technique. Glob Spine J 10:647–656 10.1177/2192568219877878 PMC7359687 32677563

[33] Menezes AH (2012) Craniocervical fusions in children. J Neurosurg Pediatr 9:573–585 22656246 10.3171/2012.2.PEDS11371

[34] Shirley ED, Demaio M, Bodurtha J (2012) Ehlers-Danlos syndrome in orthopaedics: etiology, diagnosis, and treatment implications. Sports Health 4:394–403 23016112 10.1177/1941738112452385 PMC3435946

